# Covid-19 in Italy: A Lesson to be Learned

**DOI:** 10.1055/s-0040-1713410

**Published:** 2020-06

**Authors:** Gabriele Tonni, Edward Araujo Júnior

**Affiliations:** 1 Prenatal Diagnostic Service, Department of Obstetrics and Gynecology, AUSL Reggio Emilia, Italy; 2 Department of Obstetrics, Escola Paulista de Medicina, Universidade Federal de São Paulo, São Paulo, SP, Brazil; 3 Medical course, Universidade Municipal São Caetano do Sul, Bela Vista Campus, São Paulo, SP, Brazil

Dear Editor

The new coronavirus (COVID-19) was demonstrated to be circulating in Italy before February 20, 2020, which was the date of the announcement of the first patient infected with COVID-19 in Italy. Hospital-acquired infection is a major concern and might represent the primary source of human-to-human transmission. As of March 27^th^, 9% of physicians had been infected (4,824 doctors, of whom 1,600 were in severe condition and 46 were dead) according to the Italian Superior Institute of Health. A total of 9 to 11% of proven infected patients required admission to the intensive care unit (ICU). It has been estimated that the peak of the COVID-19 outbreak will likely occur by the end of April, and if this exponential trend continues, a possible dramatic scenario of 30,000 new infected people per week might be observed, requiring more than the current maximum number of ICU beds that the Italian Health System can provide.^1^ However, the percentage of healthcare givers who have been infected with COVID-19 is somewhat severely underestimated because a policy to screen all physicians and nurses regardless of clinical symptoms has not been required by the health authorities.

Hypotheses are being made for a possible explanation of this dramatic surge of infected people observed in Italy, particularly in the northern regions, such as Lombardy, Veneto, and Piedmont. The high population density in these territories, the high industrialization rate for the number of inhabitants and severe pollution dust (similar to that of the Hubei region, in China), the lifestyle, and the role of hospitals as central care facilities might have contributed/favored the spread of the virus, together with the fact that Italy has the second highest elderly proportion of the population worldwide. As of March 31^st^, no such screening program has been activated throughout Italy even though a one-hour diagnostic test has become available. Consequently, the Superior Institute of Health has estimated that 11,252 healthcare givers have been infected, including 105 deaths of doctors and nurses.^2^ Furthermore, the Italian State Account has released a legal opinion to the Italian Federation of General Practitioners (Fimmg, in the Italian acronym) regarding an amendment to article 5.1 of the Italian Law decree n. 18 of March 17, 2020^3^ which was registered at the Budget Commission of the Italian Senate. Secondary to this unexplained decision, Fimmg has submitted an application to the Guarantee to proceed with the closure of Italian general practitioners' offices because their activities are not included within the essential level of assistance.

Time will tell if the forced lockdown of the population and adherence to guidelines will be able to prevent a higher rate of human-to-human transmission (R0 currently ranges from 2.8–3.2) by limiting the pandemic outbreak of the virus, thus decreasing both positive and severe cases and the associated mortality.

The reform of Title V of the Italian Constitution in 2001 transferred public health governing from the government to the regions, resulting in nonuniform management between the 21 regions and, thus, generating inequalities between citizens. To prevent multiple and sometimes contradictory regulations and management, which can be dramatically harmful to the public health, particularly in cases of epidemics/pandemics, the general management of public health should be directed back to the central government, specifically to the Ministry of Health.

Nonetheless, contradictory announcements and declarations of reputed virologists to the media have resulted in confusing social messages, which is a concern. Notwithstanding, it is one author's opinion (G. T.) that the lack of screening tests between doctors/nurses and citizens accessing healthcare facilities, in addition to the classification as asymptomatic rather than possible presymptomatic patients, might have additionally contributed to COVID-19 diffusion in hospital settings and in general practitioners' offices, with the highest mortality rate registered in these places.

A policy of prevention strategies for the entire population based on the use of individual protection devices and the observation of guidelines/protocols may have been started soon at the onset of the epidemic. In fact, from the time of the onset of the COVID-19 epidemic/pandemic in Italy, 15 decrees have been published by the Office of the President of the Council of Ministers, which have been difficult for institutions and citizens to apply.

As of April 4, 2020,^4^ the Lombardy region has released an order that made the use of individual protection devices mandatory for the entire population, not only for healthcare professionals but also for workers; this measure has not been undertaken previously.

Epidemiological and public health analyses of what has occurred in Italy may help prevent a similar scenario from occurring in other countries, even if the World Health Organization has declared SARS-CoV-2 a world pandemic.
